# Safety hazards in clinical practice of seclusion in psychiatric care

**DOI:** 10.1192/j.eurpsy.2022.708

**Published:** 2022-09-01

**Authors:** J. Varpula, M. Välimäki, T. Lantta, J. Berg, P. Soininen, M. Lahti

**Affiliations:** 1 University of Turku, Department Of Nursing Science, Turku, Finland; 2 Central South University, Xiangya School Of Nursing, Changsha Hunan, China; 3 Turku University of Applied Sciences, Health And Well-being, Turku, Finland; 4 Helsinki University Hospital, Department Of Psychiatry, Helsinki, Finland

**Keywords:** Safety and Security, Safety Hazards, seclusion

## Abstract

**Introduction:**

Seclusion is part of the clinical practice in European psychiatric hospital care with the aim to maintain the safety of patients and staff. Adverse events and harm have been reported for patients and staff resulting from seclusion. Safety hazards, which are the prerequisite of adverse events, can be identified using video observation methods. Identifying safety hazards can be used to prevent adverse events and improve the quality of psychiatric care.

**Objectives:**

To identify safety hazards during seclusion in psychiatric hospital care.

**Methods:**

Descriptive design with non-participant video-observation of seclusion care practice. Data consisted of video recordings (n = 36) from six wards of one psychiatric hospital in Finland. The data were analysed with inductive thematic analysis.

**Results:**

Clinical practice of seclusion included safety hazards stemming from the actions of patients and staff. Patients’ actions were as follows: aggressive behaviour, attempting to escape, precarious movements, preventing the visibility of staff, exposing themselves to contamination, and falls during seclusion. Staff actions included: leaving dangerous items to seclusion, issues in the administration of medication, performing physical and mechanical restraints in unsecure way, and precarious movements and postures.

**Conclusions:**

According to our results, the use of seclusion has safety hazards that can result in harm for patients and staff. To improve the quality and safety of seclusion in clinical practice, the guidelines, practices, and staff training need to consider the various safety hazards. While the work in Europe to abolish the use of seclusion is still in progress, this topic requires attention in clinical practice, education, and policymaking.

**Disclosure:**

No significant relationships.

